# Incidence of post-traumatic stress, anxiety and depression symptoms in patients and relatives during the ICU stay and after discharge

**DOI:** 10.1186/cc11104

**Published:** 2012-03-20

**Authors:** R Fumis, P Martins, G Schettino

**Affiliations:** 1 Hospital Sirio-Libanes, São Paulo, Brazil

## Introduction

To study the incidence and predictors of post-traumatic stress, anxiety and depression symptoms in medical and surgical patients and relatives during the ICU stay and at 30 and 90 days post ICU discharge.

## Methods

A prospective study of 72 patients and 99 family members that completed the Hospital Anxiety and Depression Scale during the ICU stay and at 30 and 90 days after discharge. The Impact of Event Scale at 30 and 90 days after ICU discharge was used to evaluate post-traumatic stress disorder (PTSD).

## Results

The prevalence of symptoms of anxiety, depression or both in patients during the ICU stay was 10%, 2.8% and 6.9% respectively. Among family members prevalence was 17.3%, 6.5% and 14.4% respectively, and was significantly higher compared to patients (*P *= 0.034). PTSD symptoms were present in 39.8% and 32.7% of family members respectively at 30 and 90 days after discharge. Among patients symptoms were significantly lower (*P *< 0.001). Factors associated with symptoms of anxiety and depression during the ICU stay in a multivariate model included patient-related factors as SAPS 3 (OR 1.1, 95% CI 1.01 to 1.24) and length of family member stay in the ICU (OR 1.39, 95% CI 0.89 to 2.16) and family-related factors as female gender (OR 5.43, 95% CI 0.67 to 43.8) and oncologic diagnosis (OR 0.25, 95% CI 0.05 to 1.31). The multivariate model also identified patient age (OR 0.97, 95% CI 0.93 to 1) and oncologic diagnosis (OR 0.27, 95% CI 0.09 to 0.79) associated with symptoms of post-traumatic stress after discharge among family members.

## Conclusion

At least one-third of family members visiting patients in the ICU suffer from symptoms of anxiety, depression or both. The level of post-traumatic stress symptoms in family members was high after ICU discharge. Depression, anxiety and post-traumatic stress symptoms were higher among family members compared to patients. Female gender and oncologic diagnosis were strongly associated with depression and post-traumatic stress. Further actions might be adopted to diminish the incidence of these disorders.

## References

[B1] Myhren H Crit Care 2010 14 R14 10.1186/cc8870 20144193 PMC2875529

[B2] Pochard F Crit Care Med 2001 29 1893 1897 10.1097/00003246-200110000-00007 11588447

[B3] Fumis R Intensive Care Med 2009 35 899 902 10.1007/s00134-009-1406-7 19183953

